# Overlooked Hoffa Fracture in a Patient With Neurofibromatosis-1

**DOI:** 10.5435/JAAOSGlobal-D-17-00060

**Published:** 2017-11-23

**Authors:** Martin Missmann, Karl Peter Benedetto

**Affiliations:** From the Department of Senior Consultants, AUVA, Austrian Workers' Compensation Board, Innsbruck, Austria (Dr. Missmann), and the Department of Trauma Surgery, Hospital Hochrum, Rum, Austria (Dr. Benedetto).

**Keywords:** Hoffa fracture, Femoral fracture, Neurofibromatosis-1, High-velocity trauma, Osteopenia, Skeletal deformities

## Abstract

Hoffa fractures are rare fractures of either one or both femoral condyles in the coronal plane. They usually occur as a result of high-velocity, high-energy trauma with axial compression of the lower limb and a typically flexed position of the knee. The lateral condyle is affected more frequently. Diagnosis of a coronal fracture is often missed in conventional radiography, so additional CTs of the knee are highly recommended in unclear cases. Because of permanent shearing forces, fracture healing is unlikely without surgical treatment. In the case we present here, the Hoffa fracture occurred after minimal trauma and was associated with an ipsilateral patellar dislocation and a meniscal tear. The fracture was initially overlooked. Bone quality was affected by a preexisting neurofibromatosis-1 condition.

## Neurofibromatosis-1

Neurofibromatosis-1 (NF1) is a common neurocutaneous condition with an autosomal dominant pattern of inheritance.^1^ It is caused by mutations of the *Nf1* gene and has an incidence of about one in 3,000.^2^ The *Nf1* gene is localized in the pericentric region of chromosome 17. The gene product, neurofibromin, functions as a rat sarcoma GTPase-activating protein, and the *Nf1* gene has been classified as a tumor suppressor.^3^ Neurofibromin is required for maintaining an appropriate sensitivity to growth factors. Dysfunction of the *Nf1* gene may lead to abnormal cellular differentiation and cellular dysfunction.

As a result of this, patients with neurofibromatosis are predisposed to a variety of tumors, typically of the peripheral and central nervous systems, and to skeletal abnormalities. Such abnormalities include osteoporosis, scoliosis and other skeletal deformities, pseudarthrosis, and impaired fracture healing. Orthopaedic problems caused by anterior bowing of the tibia, pseudarthosis, scoliosis, and reduced bone stability are common.^1,2,4^ Other clinical manifestations of NF1 are so-called café-au-lait patches, skin-fold freckling, Lisch nodules, and cutaneous neurofibromas. These manifestations are present in 85% to 99% of patients.

## Hoffa Fractures

Hoffa fractures are fractures of the distal femur in the coronal plane (OTA classification 33-B3) and can affect either the lateral or the medial or, rarely, both condyles. Unicondylar fractures of the lower end of the femur are uncommon injuries and occur approximately in 82% of cases in the sagittal plane and in 18% of patients in the coronal plane.^5^ Coronal fractures of femoral condyles are rare injuries and usually result from high-velocity, high-energy trauma.^6^ The probable mechanism of injury is axial compression to the knee, with transmission of the ground reaction force through the tibial plateau to the posterior femoral condyles when the knee is flexed >90°. The physiological valgus causes an abduction component, which explains the higher frequency of lateral condyle fractures.^7^ It is yet not clear whether the direct impact on the flexed knee alone or an axial impact combined with simultaneous vertical shear and twisting forces is the main injury pattern.^6^

Initial standard AP radiographs may be unremarkable because the fracture can be obscured by the intact anterior part of the condyle and the fracture is perpendicular to the radiographic beam.^6^ Therefore, fractures are missed in conventional radiography in up to one-third of cases. Because cases of malunion and nonunion have been reported when Hoffa fractures have not been managed surgically, it has become common that patients with Hoffa fractures need to undergo surgical treatment. Open reduction and internal fixation are recommended^8^; the treatment should follow early after the accident. Fractures of the lateral condyle carry a risk of malalignment and of degenerative joint diseases; osteonecrosis is a potential complication.^5^

## Case Report

**Figure 1:**
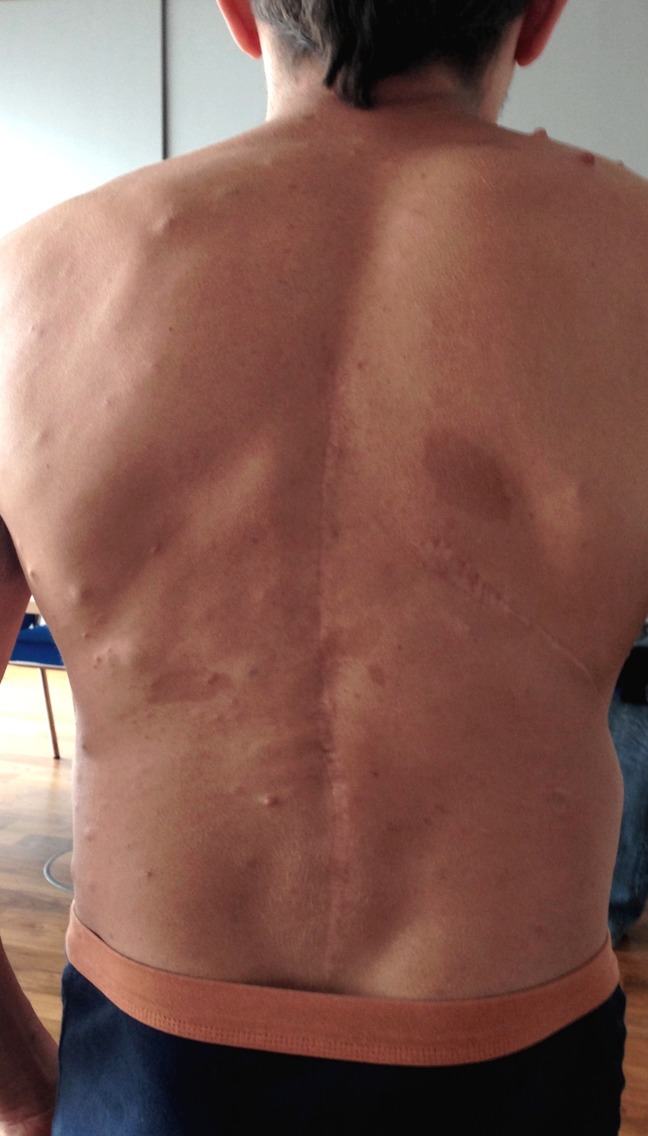
The patient with clinical signs of neurofibromatosis-1: short stature, thoracolumbar kyphosis, and scoliosis. He underwent spinal surgery twice because of reduced bone quality. Multiple café-au-lait macules and fibromas are visible.

**Figure 2:**
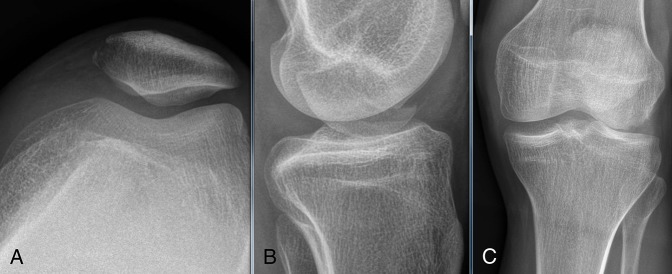
**A,** Axial radiograph of the left patella. **B,** Lateral radiograph of the left knee. **C,** AP radiograph of the knee.

A 28-year-old man presented at a traumatic surgery unit in an outlying hospital, where an external rotation of the left leg and a patellar dislocation were detected. Examination also revealed clinical signs of a neurofibromatosis-1 (Figure 1). The patient had stumbled doing his work as a cook and had fallen on his flexed left knee. Conventional radiography of the knee demonstrated an osteochondral flake near the medial patellar margin, whereas the femoral fracture remained unnoticed (Figure 2). After reposition of the patellar dislocation, the joint was stabilized with an orthosis, and the patient was sent home.

**Figure 3:**
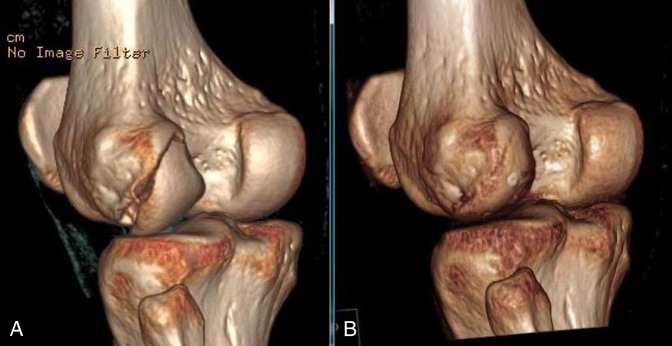
**A** and **B,** Three-dimensional rendering of the Hoffa fracture before and after surgical treatment.

Two days later, because of increasing pain and swelling of the left knee, his family doctor arranged further radiographic examinations with MRI and CT scans, revealing the Hoffa fracture. The patient was then referred to a medical center for traumatic surgery. Surgical treatment consisted of diagnostic arthroscopy, open reduction of the fracture, and internal fixation with three 40-mm headless compression screws (Figure 3). The screws were placed in posterior to anterior and caudal to cranial directions. The anterior horn of the lateral meniscus was fixed to the joint capsule using FiberWire 2-0, and the ruptured retinaculum was fixed to the medial patellar margin with two Mitek anchors. A 3 × 3 cm tumor close to the articular capsule turned out to be a lipoma and not, as initially suspected, a neurofibroma.

After surgery, the knee was stabilized with a knee orthosis, and partial weight-bearing was prescribed for 10 weeks. Fracture healing was regular. In <4 months after the accident, the patient returned to work without any orthopaedic therapeutic appliances. In this case, the occurrence of a femoral fracture after a low-velocity trauma was probably influenced by reduced bone quality because of an underlying NF1, but NF1 did not influence fracture healing. Two years after the operation, the range of motion of both knees was unlimited, and no osteoarthrotic changes were noticed.

## Discussion

Manfredini et al^7^ studied 19 subjects with unicondylar fractures of the femur; associated lesions were found to be frequent, such as other femoral fractures, patellar fracture, and anterior cruciate ligament rupture. Although in most cases, Hoffa fractures occur as a result of high-velocity trauma,^6,7,8^ Vaishya et al^9^ presented the case of a Hoffa fracture with ipsilateral patellar dislocation resulting from a household trauma. Comparable to our patient, the patient reported by Vaisha et al^9^ was first seen in a local hospital, where the patellar dislocation was treated while the Hoffa fracture was initially missed by the radiologist.

Pathologic tissue resulting from neurofibromatosis rarely affects a joint directly. In our patient, a tumor adjacent to the joint capsule turned out to be a lipoma after histopathologic examination, containing no tissue of a neurofibroma. Bone deformities of the lower limb in patients with NF1 have been described in several reports, in particular, congenital anterior bowing of the tibia and congenital pseudoarthrosis of the tibia. Skeletal dysplasia has also been reported in patients such as ours. Because of a pronounced thoracolumbar kyphoscoliosis and a reduced bone density, he had to undergo extensive surgery 10 years before the reported accident.

Bone tissue is a composite of a collagenous framework, integrated small proteins, and a mineral phase with carbonated hydroxyapatite. Defects in any of those components can cause low bone mass or diminished bone quality, or both. Poor bone quality is a known risk factor for fractures. NF1 was found to be associated with reduced bone mineral density. Contemporary studies reveal that about 48% of subjects with NF1 show osteopenia and 25% to 28% show osteoporosis.^2,10^ In our patient, diminished bone quality had been verified even before the accident. This might explain the occurrence of a Hoffa fracture after a low-velocity accident.

Fracture risk is also evidently age dependent. In a study by Heervä et al,^2^ there was no increase in fracture risk of NF1 patients aged 17 to 40 years compared with control subjects without NF1. The authors speculate that bone strength would be the highest in this age group of patients with NF1, and subsequently bone mineral density would worsen with aging. Fracture risk was evaluated in children aged 3 to 16 years with NF1, showing a relative risk ratio for fractures of 3.4,^2^ whereas fracture risk of adults with NF1 older than age 40 years was about fivefold higher than in the control group. This is in contrast to the young adult patient mentioned here. Impaired fracture healing is frequently observed in patients with NF1. However, in the above-mentioned study by Heervä et al^2^ with 460 patients with NF1, all but one of the 60 fractures had healed. These results are in accordance with the actual case, where no delayed fracture healing was observed.

In a study by Bel et al^5^ with 163 distal femoral fractures, of which 18% were Hoffa fractures, 50% of patients reported moderate pain. Osteoarthritis was found in 12% of patients and was associated with malunion. Valgus-varus deformity was found in 10% cases, flexion-recurvation deformity in 5%, and an AP or lateral articular surface step-off in 12%. In our patient, currently no osteoarthritis and no joint deformation has occurred.

## Conclusion

Hoffa fractures are unusual fractures and might initially remain undiagnosed. They normally result from high-velocity trauma but may also occur after minor trauma in subjects with diminished bone quality. This case report shows that knowledge about rare injuries and genetic disorders, such as neurofibromatosis-1, is important in helping to find a complete diagnosis and to provide an adequate therapy.
